# Evaluating web-based cognitive-affective remediation in recent trauma survivors: study rationale and protocol

**DOI:** 10.1080/20008198.2018.1442602

**Published:** 2018-03-05

**Authors:** Naomi B. Fine, Michal Achituv, Amit Etkin, Ofer Merin, Arieh Y. Shalev

**Affiliations:** ^a^ Psychological Trauma Care Center, Shaare-Zedek Medical Center, Jerusalem, Israel; ^b^ Department of Psychiatry and Behavioral Sciences, Stanford University School of Medicine, Stanford, CA, USA; ^c^ Trauma Unit and Department of Cardiothoracic Surgery, Hebrew University Faculty of Medicine, Shaare Zedek Medical Center, Jerusalem, Israel; ^d^ Department of Psychiatry, NYU Langone Medical Center, New York, NY, USA

**Keywords:** Post-traumatic stress disorder, cognitive-affective remediation, neuroplasticity, executive function, emotion bias, emotional regulation, Trastorno por estrés postraumático, reparación cognitivo-afectiva, neuroplasticidad, función ejecutiva, sesgo emocional, regulación emocional, 创伤后应激障碍, 认知情感矫正, 神经可塑性, 执行功能, 情绪偏差, 情绪调节, • Protocol of early neurocognitive intervention for acute trauma.• Study rational and design.• Barriers and challenges to early computerized neurocognitive intervention: specifically; participant enrolment, efficient implementation and management of daily self-administered, web-based intervention, reliable and timely screening and in-depth assessment of survivors at significant risk of PTSD and defining control conditions.• Discussion of assessment and outcome measures.

## Abstract

**Background**: The immediate aftermath of traumatic events is a period of enhanced neural plasticity, following which some survivors remain with post-traumatic stress disorder (PTSD) whereas others recover. Evidence points to impairments in emotional reactivity, emotion regulation, and broader executive functions as critically contributing to PTSD. Emerging evidence further suggests that the neural mechanisms underlying these functions remain plastic in adulthood and that targeted retraining of these systems may enhance their efficiency and could reduce the likelihood of developing PTSD. Administering targeted neurocognitive training shortly after trauma exposure is a daunting challenge. This work describes a study design addressing that challenge. The study evaluated the direct effects of cognitive remediation training on neurocognitive mechanisms that hypothetically underlay PTSD, and the indirect effect of this intervention on emerging PTSD symptoms.

**Method**: We describe a study rationale, design, and methodological choices involving: (a) participants’ enrolment; (b) implementation and management of a daily self-administered, web-based intervention; (c) reliable, timely screening and assessment of treatment of eligible survivors; and (d) defining control conditions and outcome measures. We outline the rationale of choices made regarding study sample, timing of intervention, measurements, monitoring participants’ adherence, and ways to harmonize and retain interviewers’ fidelity and mitigate eventual burnout by repeated contacts with recently traumatized survivors.

**Conclusion**: Early web-based interventions targeting causative mechanisms of PTSD can be informed by the model presented in this paper.

## Abstract (es)

El período inmediatamente posterior a los eventos traumáticos es un período de mayor plasticidad neuronal, después del cual algunos sobrevivientes siguen con trastorno de estrés postraumático (TEPT) mientras que otros se recuperan. La evidencia señala que las deficiencias en reactividad emocional, regulación de las emociones y funciones ejecutivas más amplias contribuyen de manera crítica al TEPT. La evidencia que comienza a hacer su aparición sugiere además que los mecanismos neuronales que subyacen a estas funciones siguen siendo plásticos en la edad adulta y que la reeducación específica de estos sistemas puede mejorar su eficacia y reducir la probabilidad de desarrollar un TEPT. La administración de entrenamiento neurocognitivo específico poco después de la exposición al trauma es un desafío desalentador. Este trabajo describe un diseño de estudio que aborda ese desafío. El estudio evaluó los efectos directos del entrenamiento en reparación cognitiva sobre los mecanismos neurocognitivos que hipotéticamente subyacen al TEPT y el efecto indirecto de esta intervención en los síntomas de TEPT que surgen. Método: Describimos una justificación para el estudio, unas opciones de diseño y una metodología que implican (a) inscribir a los participantes; (b) implementar y administrar una intervención diaria autoadministrada, basada en la web; (c) detectar y evaluar, de modo oportuno y fiable, el tratamiento de los sobrevivientes que resultan elegibles; y (d) definir las condiciones de control y las medidas de los resultados. Resumimos la justificación de las decisiones que se tomaron con respecto a la muestra del estudio, el momento de la intervención, las mediciones, el seguimiento del compromiso de los participantes y el modo de armonizar y retener la fidelidad de los entrevistadores y mitigar el agotamiento debido al contacto repetido con sobrevivientes recientemente traumatizados. Conclusión: las intervenciones tempranas basadas en la web que se dirigen a los mecanismos que causan el TEPT pueden basarse en el modelo presentado en este documento.

## 背景

发生创伤事件之后的短时期内神经可塑性得到增强，在此之后有些幸存者出现创伤后应激障碍（PTSD），而有的则会康复。有证据指出，情绪反应、情绪调节和广义的执行功能损伤是造成 PTSD 的关键。越来越多的证据进一步指出，这些功能背后的神经功能在成年期依然保持可塑性，针对这些系统的再训练可能提高它们的效能，并减少出现 PTSD 的可能性。在创伤暴露后的短期内进行神经认知训练是个充满挫败的挑战。本文描述的研究设计将应对这项挑战。本研究评估了认知矫正训练对PTSD理论上的神经认知机制的直接效果，和间接干预正在出现的 PTSD 症状的效果。

方法：我们描述了研究原理、设计和方法选择，包括a）被试招募； b）实施和管理一个每天进行的自助网络干预； c）对合适的幸存者进行可靠及时的筛选和治疗评估；d） 定义控制条件和结果测量。我们列出了关于研究样本、干预时间、测量、评估被试配合度的选择背后的原理，以及如何平衡和保持面试者的真实性、通过反复联系最近经历创伤的幸存者达到减轻其倦怠的方法。

结论：本文描述了一个针对 PTSD的致病机制的早期网络干预模型。

## 1. Introduction

Post-traumatic stress disorder (PTSD) is a commonly occurring disorder that has a profound public health impact by virtue of its high prevalence, persistence, accompanying functional impairment, and elevated risk of other mental and physical disorders. Transient PTSD symptoms are frequently observed shortly after traumatic events, remit in many survivors, leaving approximately 30% of those who meet acute PTSD diagnostic criteria with chronic, unremitting PTSD (Koren, Arnon, & Klein, 2001; Perkonigg et al., 2005). The occurrence of chronic PTSD in a subset of initially symptomatic survivors suggests an underlying and persisting neurobehavioural modification.

The occurrence of PTSD has been associated with numerous neurobehavioural risk factors. Broad pre-exposure emotional vulnerability traits, such as neuroticism (Brewin, Andrews, & Valentine, 2000), low self-efficacy, and high hostility (Heinrichs et al., 2005), are robustly associated with the development of PTSD. Premorbid emotional biases, such as the tendency to pay attention to negative (sad) faces and to avoid processing of fearful faces, predicted post-stressor depressive and PTSD symptoms in soldiers deployed in active war-zone settings (Beevers, Lee, Wells, Ellis, & Telch, 2011). Biased pre-trauma emotional processing coupled with low executive functions (Aupperle, Melrose, Stein, & Paulus, 2011) and poor general cognitive functioning significantly increased the risk of PTSD following trauma exposure (Kremen et al., 2007). Numerous neurocognitive deficits have been linked with the emergence of PTSD (for a recent review see Scott et al., 2015). These include working memory, information processing speed and verbal learning, as well as impairments in short-term and declarative memory (Johnsen & Asbjørnsen, 2008; Samuelson, 2011), attention, and executive functioning (Aupperle et al., 2011; Polak, Witteveen, Reitsma, & Olff, 2012). The associations between neurocognitive deficits and PTSD were independent of concurrent head injury or substance use disorders (Wrocklage et al., 2016). Most studies to date, however, used a cross-section design and as such could not determine whether PTSD-associated neurocognitive deficits reflect pre-exiting vulnerability traits (e.g. Gilbertson et al., 2006; Vasterling & Brailey, 2005), emerging disorder attributes, or an interaction between the two.

Neurocognitive deficits have additionally been shown to negatively affect the outcome of treatment in PTSD. Greater efficiency of inhibitory function and poorer verbal memory predicted response to cognitive-behavioural therapy in PTSD (Falconer, Allen, Felmingham, Williams, & Bryant, 2013; Wild & Gur, 2008). Similarly, cognitive dysfunction negatively affected the outcome of pharmacological treatments, in which the ability to cooperate in and successfully complete treatment relies on cognitive engagement (Dunkin et al., 2000; Jaeger & Vieta, 2007). Conversely, treatment with the SSRI paroxetine resulted in a significant increase in memory functions before and after treatment among survivors with PTSD (Fani et al., 2009). Finally, cognitive deficits accurately predicted current social and occupational functioning in veterans with PTSD (Geuze, Vermetten, De Kloet, Hijman, & Westenberg, 2009) and were associated with occupational functioning and physical health-related quality of life (Wrocklage et al., 2016).

Taken together, impairments in emotional, self-regulatory, and cognitive functions may critically influence pre- and peri-traumatic processing of stressful events, development of PTSD, and treatment outcomes. Putative neural mechanisms underlying their pathogenic effect may involve a combination of a hyperactive emotional reactivity system coupled with dysfunctional regulatory circuitry in the frontal lobes (Etkin & Wager, 2007; Hayes, Hayes, & Mikedis, 2012; Pitman et al., 2012). Beyond the fronto-limbic circuitry, aberrations between and within (Sripada et al., 2012) the default mode network (DMN), salience network (SN), and the central executive network (CEN) were demonstrated in PTSD. The DMN and CEN were found to be weakly interconnected and hypoactive, hypothetically destabilized by an overactive and hyper-connected SN and a low saliency perception threshold, along with inefficient DMN-CEN modulation (Akiki, Averill, & Abdallah, 2017). Abnormalities within the CEN may underlie some of the cognitive, executive, and emotional regulatory dysfunctions in PTSD (Lanius, Frewen, Tursich, Jetly, & McKinnon, 2015).

Pertinent to our work, neurocognitive impairments in PTSD might be amenable to ‘top-down’ cognitive remediation that focuses on the therapeutic activation of higher-order systems, such as executive functions, as a way to improve neurocognitive processing in PTSD and restore CEN functioning (Lanius et al., 2015). Neurofeedback, which involves training of blood oxygenation level dependent (BOLD) response in specific brain regions (Cannon et al., 2007) or enhancing EEG activity facilitating cognitive performance (Gruzelier, 2014; Vernon et al., 2003) offers other way of therapeutically targeting cognitive domains neuroplasticity. Interventions targeting putative neurocognitive domains might be particularly efficient when applied at the early aftermath of traumatic events, a period of accelerated learning and increased brain plasticity, during which traumatic memories and their links with defensive alarm responses consolidate.

Early intervention studies have shown a long-lasting effect, along with significant constraints on implementing these interventions at early aftermath of trauma exposure and varying efficacies of specific interventions (e.g. Shalev, Ankri, Peleg, Israeli-Shalev, & Freedman, 2011). Pharmacological interventions that target the acquisition and extinction of fear responses have generally failed to prevent PTSD (Cohen et al., 2011; Hoge et al., 2012; Pitman et al., 2002; Stein, Kerridge, Dimsdale, & Hoyt, 2007). Early cognitive behavioural interventions, although often effective (Roberts, Kitchiner, Kenardy, & Bisson, 2010), do not reach many symptomatic survivors (Hoge et al., 2004; Shalev et al., 2012, 2011), are costly, and are difficult to carry out in trauma-affected areas such as war and disaster zones. Devising novel interventions to prevent PTSD is a major public health need (Berg et al., 2008).

Among such interventions, those involving neurocognitive functions are of particular interest (Wald et al., 2011). For example, a recent study has shown an improved neurocognitive functioning in four participants after executive training intervention along with transcranial direct current stimulation (tDCS) (Saunders et al., 2015).

Challenges to administering early neurocognitive interventions for PTSD include sample identification and risk prediction, interventions’ desirability acceptance and adherence, and loss of subjects to follow-up. Studies have shown that participants at low risk for PTSD recover equally with or without treatment (Roberts, Kitchiner, Kenardy, & Bisson, 2009; Shalev et al., 2011) and their inclusion in a study protocol creates a confound to early interventions efficacy estimation. Studies have also shown significant barriers to accepting early treatment and a non-random loss of subjects to follow-up reflecting early symptom severity. Neurocognitive retraining interventions add to these challenges by requiring frequent (daily) self-administered sessions with no within-session guidance or monitoring by therapists. Unlike trauma-focused therapies, they also lack an intuitive linkage with PTSD symptoms and therefore may challenge participants’ motivation and sense of relevance. Finally, they target dimensions of functioning with yet unsubstantiated effects on PTSD symptoms and thus must be optimally assessed using both primary (improved neurocognitive functioning) and secondary (PTSD symptoms) outcome measures. The protocol presented below is an attempt to address these constraints in a real-world implementation.

Our overall aim was twofold: (a) improve participants’ cognitive functioning in specific, intervention-targeted, neurocognitive functions (emotional reactivity and regulation and executive functions; Dandeneau, Baldwin, Baccus, Sakellaropoulo, & Pruessner, 2007; Etkin, Egner, Peraza, Kandel, & Hirsch, 2006; Hallion & Ruscio, 2011) and (b) reducing PTSD symptoms (secondary target). We hypothesized that gains in emotional reactivity, emotional regulation, and executive functioning might secondarily ameliorate the course of early PTSD symptoms. We powered the study to address its primary target and explore its secondary targets towards justifying confirmatory studies. This report focuses on designing and implementing these interventions in the acute aftermath of traumatic events. We outline each challenge to implementing the interventions, the required methodological choices and our way of addressing them, and the optimization of treatment design and results’ generalizability.

## 2. Methods/design

### 2.1. Methodological challenges

1. Identifying participants at risk and proper timing of interventions

The successful implementation of early interventions hinges upon addressing significant constraints and known barriers that include treatment acceptance (Hoge et al., 2004), providers’ skills and training, interventions’ optimal timing after the traumatic event, sample selection, and interventions’ inherent efficacy. Methodological challenges emanating from the above include the following: samples studied at the early aftermath of traumatic events necessarily include participants who recover spontaneously and thus effect of early interventions might be confounded by an unknown rate of *spontaneous recovery*, which the study design must be able to address; interventions’ *optimal timing* relative to the traumatic event poses another problem, where treatment administered too soon necessarily addresses numerous survivors with short-lived reactions to the trauma, but treatment provided too late may miss a critical window of opportunity; the *acceptance of early interventions* by survivors is often low and potentially biased (Shalev et al., 2011) and such bias must be measurable at studies’ termination. Solutions to these challenges have been poorly mapped, and their implementation requires explicit study-specific methodological choices, addressed below.

(2) Treatment engagement and implementing efficient web-based intervention

Trauma survivors who are likely to develop PTSD are often distressed, anxious, and preoccupied with consequences of the recent event, and with eventual changes to their lives. Successfully engaging and enrolling symptomatic and distressed participants, however, is critical to preventing a likely PTSD outcome in survivors at significant risk. This requires both clinical and interpersonal skills, and awareness of survivors’ real-world concerns. Participants’ acceptance and adherence to self-administered, web-based intervention are additionally challenging, given survivors’ distress and real-life concerns and the novelty of the self-administered interventions to both clinicians and patients (Ipser, Seedat, & Stein, 2006).

### 2.2. Addressing the challenges

#### 2.2.1. Choices regarding the study overall design

Experience from a previous large early intervention study (Shalev et al., 2012, 2011) informed many of our choices in this work. To facilitate the enrolment of participants at substantial risk of PTSD, we opted to employ skilled clinicians, thoroughly trained in the specific evaluation procedures. To evaluate a potential sampling bias, we followed all eligible participants regardless of their participation in treatment. (For example, this work could not accommodate participants without home computers and stable internet access.) To palliate for lack of therapist presence during the sessions, we implemented day-to-day tracking of participants’ adherence, delivered daily reminders to participants, and collected information about participants’ logging into the treatment modules’ site and duration of training and tasks completed. We made the training tasks attractive, playful, easily accessible, and personal (by matching tasks’ difficulties with participants’ ability and progress). Finally, we devised an electronic data capture platform capable of amalgamating different sources of data and real-time flagging of data quality problems.

### 2.3. Specific implementation choices

#### 2.3.1. Subjects

To assure a stable stream of participants at significant risk for PTSD and reach a sample size powered to address our main aims, we planned to recruit 100 adult survivors of traumatic events who were admitted to a general hospital emergency department (ED) after potentially traumatic events. Because the emergence of early symptoms provides better estimate of PTSD risk then ED distress or behaviour, we opted *not* to recruit in the ED itself and instead identified potential participants using a computerized file of ED ‘trauma’ admissions, available to the study team within hours of ED visits. Participants were to be considered for a telephone screening interview if they: (i) were 18–65 years old; (ii) were able to read and comprehend Hebrew or English (languages used in neurocognitive tasks); (iii) lived within the greater metropolitan area surrounding the hospital (and thus were able to attend successive assessments); and (iv) arrived to the ED because of one of the following: car accidents, terrorist attacks, work accidents, home accidents, burns, physical assaults, or large-scale disasters.

To reduce confounds related to concurrent disorders, we did not include: (i) survivors with open head injury or in coma upon ED arrival; (ii) survivors with known medical conditions that would interfere with their ability to give informed consent or cooperate with screening and/or treatment; (iii) survivors with chronic PTSD from previous events, current or lifetime psychotic illness, current substance abuse, suicidal risk, or mental disorders or conditions that constitute treatment priority. To minimize the interference of concurrent treatment with the study’s tasks, survivors being treated with benzodiazepines and those receiving cognitive behavioural therapy for their posttraumatic symptoms were not included in treatment but followed as well.

### 2.4. Instruments

#### 2.4.1. Psychometrics and diagnostic instruments

Those were chosen with special focus on continuity with previous research and use of repeatedly validated instruments allowing cross-studies comparison as follows:

##### 2.4.1.1. Psychodiagnostics Clinician-Administered PTSD Scale (CAPS) IV and 5 *(Blake et al., 1995; Weathers et al., 2016)*

structured clinical interview evaluating DSM-IV (CAPS IV) and DSM-5 PTSD symptom criteria on dimensions of frequency, intensity, and severity. CAPS contains explicit, behaviourally anchored questions and rating scale descriptors to enhance reliability. It yields a categorical (present/absent) notation of each of DSM PTSD criterion and a continuous symptom severity score, obtained by summing individual items’ scores. During the design of study, the DSM criteria changed. To ensure continuity with previous DSM-IV-based research, both DSM-IV and DSM-5 data were collected using a *combined DSM-IV and DSM-5 instrument* (available upon request). The Hebrew version used in this work was cross-translated and compared with the original English instruments. Internal consistency of CAPS-5 is 0.88 and test-retest reliability is 0.78 (Weathers et al., 2017).

##### 2.4.1.2. *Structured clinical interview for DSM-IV (SCID)* (First, Spitzer, Gibbon, & Williams, 1995)

structured clinical interview evaluating current and lifetime (pre-event) Axis I mental disorders. The Hebrew version of SCID has been used extensively in published PTSD research (e.g. Hirschfeld et al., 2000; Rohde, Lewinsohn, & Seeley, 1997).

##### 2.4.1.3. Psychometrics Post Traumatic Checklist (PCL) *(Blevins, Weathers, Davis, Witte, & Domino, 2015)*

20-item self- or interviewer-administered inventory that indexes PTSD symptoms. The PCL-5 was adapted from the original PCL to map directly onto PTSD’s symptom criteria of the DSM-5 and was cross-translated to Hebrew. The original PCL is correlated with CAPS (*r* = 0.929). The PCL-5 internal consistency is 0.94 and its test-retest reliability is 0.82.

##### 2.4.1.4. *Peritraumatic Distress Inventory (PDI)* (Brunet et al., 2001)

13-item self- or interviewer-administered inventory to assess the immediate responses to traumatic events. PDI scores obtained shortly after trauma exposure positively correlate with the occurrence of PTSD (Nishi et al., 2010). The internal consistency for the PDI is 0.75 and test-retest reliability is 0.74. The Hebrew version was cross-translated.

##### 2.4.1.5. Beck Depression Inventory (BDI) *(Beck, Steer, & Brown, 1996)*

21-item self-report instrument that evaluates the severity of current depressive symptoms. The internal consistency of BDI is 0.93 and test-retest reliability is 0.93.

##### 2.4.1.6. Beck Anxiety Inventory (BAI) *(Beck, Epstein, Brown, & Steer, 1988)*

21-item self-report instrument expressing common symptoms of anxiety. The internal consistency of BAI is 0.92 and test-retest reliability over one week is *r(81*) = 0.75.

#### 2.4.2. Neurocognitive measures

Neurocognitive tests were administered through WebNeuro (Silverstein et al., 2007), a web-based cognitive assessment battery previously validated against traditional neurocognitive tests. To reduce the effect of learning between testing sessions, we used two WebNeuro versions that include the use of different stimuli and trial sequences. WebNeuro accommodates both Hebrew and English languages. To standardize testing conditions, all tests were taken in our laboratory in the receiving hospital rather than participants’ homes. Specific neurocognitive tests include the following:

Executive functions: *N-back Task (working memory)*: Participants indicated whether the current letter on the screen matches a letter presented n-steps back. Successful performance on this task requires constant updating of memory storage and focus. *Trail Making Task (task shifting)*: Participants were instructed to connect letters and numbers sequentially while alternating between letters and numbers (e.g. 1-A-2-B, etc.). Successful performance on this requires continuous shifting back and forth between task sets, while keeping in mind the previously connected items. *Stroop Task* (Stroop, 1935) *(focus/inhibition)*: Participants were presented with colour names printed in either matching or mismatched colours (e.g. the word RED printed in green ink). The task is to indicate the ink colour. Successful performance on this task requires participants to inhibit a reaction to the word and prefer the ink colour. *Maze Task (composite executive function)*: Participants were instructed to memorize a complicated sequence of flashing dots, then re-enact it three times in a row without errors. Successful performance on this task requires storing information in working memory, resist impulsive moves when re-enacting the sequence, and doing this as fast as possible.

Emotion regulation: *Emotional conflict task*: Participants were asked to identify whether a facial expression was fearful or happy while ignoring an overlaid emotional word. Facial expression photographs were drawn from the set of Friesen and Ekman (1976). The task consists of 74 event-related presentations (1 s), with a varying inter-stimulus interval of 2–4 s (mean 3 s), in a pseudo-random order, counterbalanced across trial types for expression, word, and response button. There are neither direct repetitions of the same word with varying face distractors, nor direct repetitions of exact face–word–distractor combinations, in order to avoid negative and repetition priming effects, respectively (Mayr, Awh, & Laurey, 2003). Behavioural data consist of reaction time (RT) for correct trials (excluding error and post-error trials) and accuracy. *Emotion recognition and attention bias test*: This consists of two phases. The first phase measures reaction time of recognizing emotional facial expressions (happy, fearful, sad, angry, disgusted), followed by a surprise recognition task 10 minutes later where the focal task involves measuring reaction times for recognition of previously presented individuals compared to newly presented individuals. The test measures both emotional labelling accuracy and the interference of facial emotional expressions of emotion with recall accuracy.

### 2.5. Intervention

Participants were blindly allocated to either a neurobehavioural training group or one of two control groups. In the training group, all tasks had ‘game-like’ features making them visually engaging and motivating, providing feedback about performance. The tasks specifically targeted executive function (e.g. working memory, task switching, resisting interference) and emotional reactivity and regulation. The training tasks were based on classic paradigms used in cognitive neuroscience and executive function batteries. The cognitive training targets emotion regulation and emotional reactivity by instilling emotional bias toward positive stimuli and emotion recognition is trained by tasks that enhance the ability to detect and label facial expressions.

The two control groups were designed to (a) control for specific neurocognitive tasks and (b) control for eventual beneficial effect of performing active computer ‘games’ and thus being distracted from post-traumatic preoccupations. The first group was engaged in web-based tasks with similar visual appeal that do not address specific neurobehavioural domains such as card games, visual search tasks, and different kinds of matching tasks. Time-on task but not performance data were collected on the control tasks. The second control group consisted of visually appealing reading tasks whose contents were limited to emotionally neutral topics (e.g. nature, geography). It was meant to control for the engaging aspects of both neurocognitive retraining and control computer games.

### 2.6. Procedure

A member of the research team identified potentially trauma-exposed ED patients using the ED medical records. Within 7–14 days after potential trauma exposure, the identified individuals were contacted by telephone. After verbal consent, the PCL and PDI were administered to assess risk of PTSD development. Those who met PTSD symptom criteria (except the one month duration) and did not meet any of the exclusion criteria received verbal information about the study and were subsequently invited to participate in a pre-intervention assessment that included administration of CAPS (Blake et al., 1995; Weathers et al., 2016), SCID (First et al., 1995), and the WebNeuro battery (Silverstein et al., 2007) (T1). The same assessment followed the completion of the intervention or control groups at three months (T2) and at six months (T3).

The intervention consisted of daily 30-minute sessions for 30 days up to five weeks. It included a combined regimen of Lumosity cognitive training games and MyBrainSolutions emotional bias training previously applied in major depression and generalized anxiety disorder population (Gyurak, Gross, Chan, & Etkin, 2013). On each training day, participants were instructed to train each task for 3–4 minutes, and were given eight tasks, chosen at a random sequence within each category (categories are: focus/inhibition, working memory, task shifting, emotion recognition/resisting distraction, positivity bias) or 10 control tasks chosen at random. Specifically, cognitive control (focus/inhibition) was trained with the ‘Eriksen Flanker task’, a response inhibition test used to assess and train suppression of responses that are inappropriate in a particular context. The stimuli included a central object with distracting stimuli. Participants were to indicate the direction of objects (schematic birds or arrows) on the screen amid other distractor objects (schematic trees or flanking arrows). Another focus/inhibition task included selecting constantly moving fish, continuously remembering which fish were already selected. Working memory was trained using a speeded version of the ‘N-back task’ that was comprised of remembering series of objects, and a spatial working memory task that included remembering locations of objects. Task shifting was trained with the ‘Plus-Minus task’: participants were instructed to either add or subtract numbers from numbers displayed on the screen. Depending on the location of the information on the screen, participants’ task was to indicate whether their answer was even or odd. Spatial task shifting was trained via matching pieces of puzzle with geometric shapes based on orientation. Positivity bias was trained by selecting a positive face out of a matrix of faces and looking at fast moving stimuli (faces) while selecting the smiling faces. Frustration tolerance was measured by increasing level of difficulty and speed in order to provoke frustration and account for response pattern reaction to difficult arithmetic exercises.

**Figure 1.**
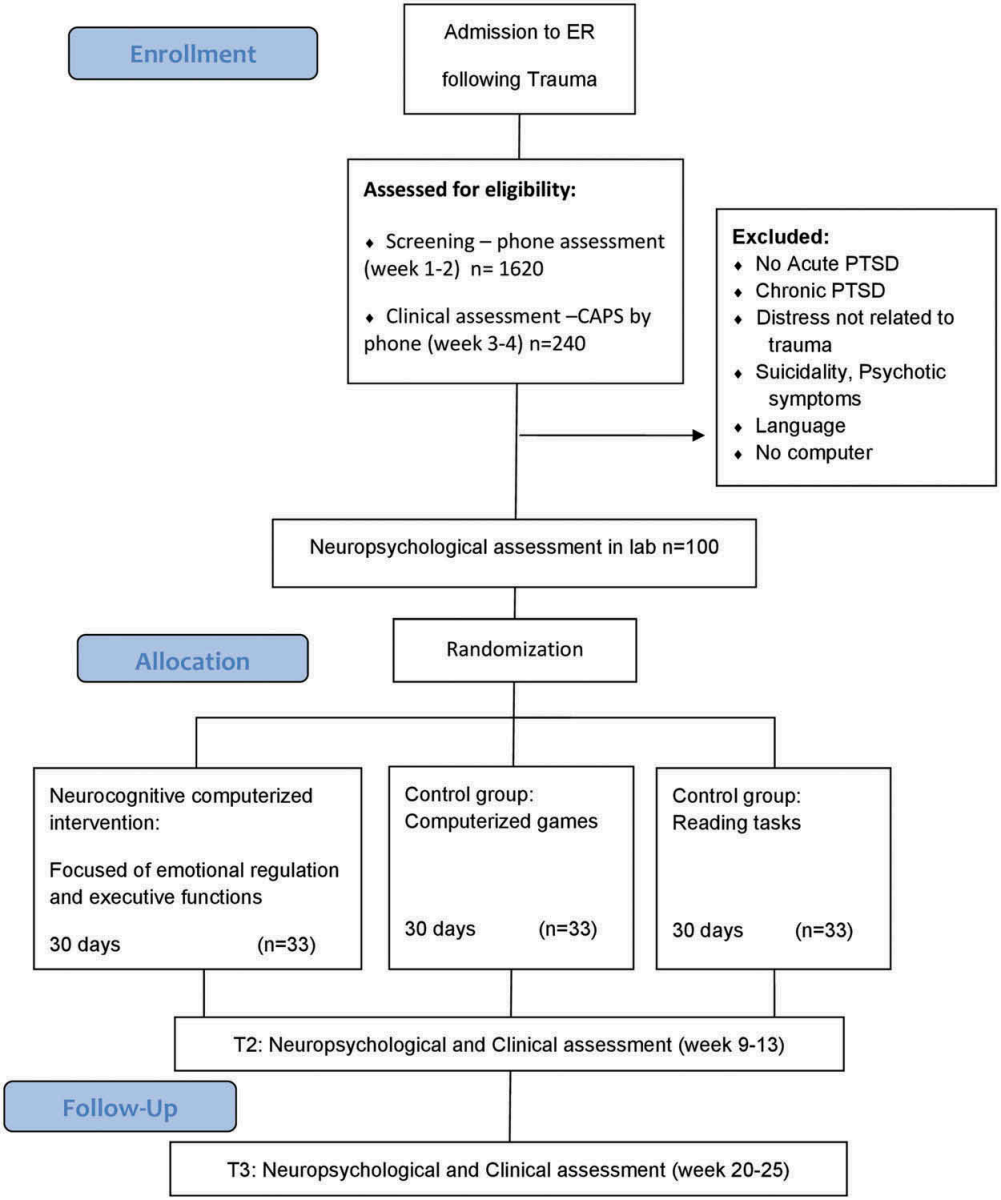
Clinician-Administered PTSD Scale, neuropsychological assessment, WebNeuro.

All tasks were designed to be dynamic, adaptive, and continually engaging, such that they increase in difficulty level as performance improves. Examples of such adapted features include: shapes moved faster, previous shapes faded out (working memory), visual complexity increased, rules faded out and changed (visual task shifting), number of stimuli increased, presentation times decreased (positivity bias). Learning in the training exercises was calculated from daily reaction times and accuracies using linear mixed models on daily performance indices to derive within-subject slope. This approach allows greater variety of training experiences and diminishes boredom. Our custom web-monitoring interface allowed us to closely track completions, minimize data loss, and maximize compliance. Participants were randomized to either treatment or control groups with balanced allocation for gender (see Figure 1).

### 2.7. Data analysis

For all analyses, we examined group × time interactions using linear mixed models to model change over time for neurocognitive and clinical measures in an intent-to-treat framework. To evaluate attrition, we compared the study completers vs. drop-outs to test if they differed in baseline characteristics such as age and gender. We assessed the impact of missing data via a repeated measures mixed effects model by running two different analyses, one with imputed missing data and another one with existing data. In the context of our mixed models analysis, we examined individual differences at baseline which predicted the efficacy of the intervention on primary and secondary outcomes. Results were expressed as differences in mean scores between the study groups with 95% confidence intervals. *P*-values <.05 were considered to indicate statistical significance.

## 3. Discussion

This work presents a study design that investigated the direct effects of targeted, cognitive-affective remediation training on neurocognitive mechanisms that underlay post-traumatic psychopathology (primary outcome) and explored the amelioration of emerging PTSD symptoms (secondary outcome) at the early aftermath of trauma exposure. This publication discussed and made explicit several methodological and practical challenges associated with providing early, innovative, web-based, neurocognitive intervention for trauma survivors at high risk of developing PTSD and evaluating its primary and secondary effects. The proposed intervention addressed neurobehavioural functions implied in the aetiology of PTSD (executive function, emotion reactivity, regulation), that hypothetically underly recent survivors’ recovery from early symptoms. As such, the intervention was seen as optimally suited for implementation during the first weeks that follow trauma exposure.

The proposed intervention did not directly target PTSD symptoms or cognitions, but rather specific neurocognitive functions hypothetically leading to PTSD. This approach is in line with the recent National Institute of Mental Health (NIMH) policy, according to which studies of new technologies must define a primary target and start by demonstrating the intervention’s ability to engage that target.

Special challenges to this work included participants’ motivation and willingness to self-administer the training exercises and their ability to follow the treatment protocol after a short introductory session. Consequently, participants’ adherence to treatment and compliance with daily work were specifically assessed. This was done by combining an automatic recording of treatment attendance and sessions’ length, using the intervention’s provider monitoring, with in-person follow-up and contact with the participants.

Because the quality of longitudinal studies of trauma survivors hinges upon reaching out to and engaging distressed trauma survivors during an early period of presumed higher neuronal plasticity, we combined rapid outreach and brief screening assessments (to reduce the burden on more resource-demanding clinical and neurocognitive assessments) with in-depth assessments by qualified professionals – both within a reasonably short time after trauma exposure. Based on that experience we suggest that the critical enrolment interviews, which ultimately determine the quality of samples enrolled, be conducted by trained clinicians who can optimally identify survivors at high risk and whose work combines clinical competency, instrument literacy, and optimal communication skills. We also recommend that clinicians and other interviewers engage in frequent (weekly, if possible) consensus and diagnostic group discussions that can mitigate some of the burden on interviewers and reduce burnout while at the same time allowing in-depth discussion of cases in doubt and higher quality of decisions made.

To assure studies’ generalizability, and account for sampling bias due non-random attrition, it is advisable to maintain participants unable or unwilling to attend treatment in a follow-up assessment without intervention, and longitudinally assess initial and time-dependent differences between these individuals and those who attended treatment. Proper, supportive training in study procedures and timely and open communication with accessible team members, allowed participants to clarify issues as they present themselves (e.g. software issues, difficulties with instructions) and reduce technical and motivational barriers to study adherence.

Lastly, the training interventions themselves were conducted in a dose and a progression that matched to each participants’ ability, and adapted tasks’ difficulty to the participants’ learning rate. Importantly, however, while proper methodology cannot eliminate attrition and dropout, it might assure that eventual attrition better represents inherent difficulties related to interventions’ content that better represent true parameter of interventions’ effectiveness.

The current study protocol is limited by not accounting for several risk factors for cognitive malfunction and PTSD. Specifically, early life trauma is associated with impairment in cognitive and emotional functions (Burri, Maercker, Krammer, & Simmen-Janevska, 2013; Pechtel & Pizzagalli, 2011) across studies exploring genetic (Klengel et al., 2013), psychosocial (Ogle, Rubin, & Siegler, 2013), and gene by environmental contributions (Binder et al., 2008). Early life trauma was not evaluated in this work, thereby limiting our ability to address a potential source of cognitive impairment.

Similarly, dissociation was not properly evaluated in depth (i.e. CAPS de-realization and de-personalization items were the only dissociative features captured in this work). Dissociation’s effects on neuropsychological dysfunction is a recurrent finding (McKinnon et al., 2016; Parlar, Frewen, Oremus, Lanius, & McKinnon, 2016), most often linked to PTSD stemming from childhood trauma (De Bellis, Woolley, & Hooper, 2013; Rivera-Vélez, González-Viruet, Martínez-Taboas, & Pérez-Mojica, 2014), but also documented in veterans with PTSD (Roca, Hart, Kimbrell, & Freeman, 2006). Dissociative types of PTSD might have a different underlying neural mechanism and specific deficits in emotional learning (Ebner-Priemer et al., 2009; Lanius et al., 2010).

As such, the proposed design does not exhaust many putative factors affecting responses to cognitive intervention. Further research should also include subjective measures of cognitive improvement, as those might not be in line with objective measurements.

## Availability of data and materials

Data currently compiled and QA’d for analyses. Will be available upon demand once brought to maturity by contacting the study PI.
